# Intracranial extension of orbital inflammatory pseudotumor: a case report and literature review

**DOI:** 10.1186/s12883-016-0550-2

**Published:** 2016-02-29

**Authors:** Enrico Tedeschi, Lorenzo Ugga, Ferdinando Caranci, Francesca Califano, Sirio Cocozza, Giacomo Lus, Arturo Brunetti

**Affiliations:** Department of Advanced Biomedical Sciences, Federico II University of Naples, Naples, Italy; Department of Clinical and Experimental Internal Medicine “F. Magrassi and A. Lanzara”, Second University of Naples, Naples, Italy

**Keywords:** Orbital inflammatory pseudotumor, Magnetic resonance imaging, MRI, Orbital mass, Intracranial extension

## Abstract

**Background:**

Orbital inflammatory pseudotumor is a rare inflammatory condition of unknown cause that may extend intracranially, usually as a dural-based infiltrate. Here we report the first case of orbital pseudotumor presenting with intra-axial Magnetic Resonance Imaging (MRI) changes.

**Case presentation:**

A 57-year-old white female, with a 3-month history of headache and right palpebral edema, presented with marked right temporal lobe edema with ominous MRI appearance, and ipsilateral alterations of orbital and periorbital structures. Following steroid therapy, both intracranial and orbital involvement dramatically improved.

**Conclusion:**

Orbital inflammatory pseudotumor with chronic inflammation may infrequently present with intracranial involvement, mimicking more aggressive diseases, even showing intra-axial enhancement after i.v. contrast administration in brain MRI. Awareness of this possibility may help neurologists to choose the appropriate therapeutic approach.

## Background

Inflammatory pseudotumor is a rare benign condition of unknown cause, characterized by unencapsulated mass-like aggregates of myofibroblastic spindle cells and inflammatory cells, including lymphocytes [1]. It has been described in almost any location, with no age- or sex- preference, although most commonly in the lung and orbit [2]. Orbital inflammatory pseudotumor ranks third after Graves’ and lymphoproliferative diseases among the most common orbital pathologies [3], and accounts for approximately 5–8 % of all orbital masses. It may present as an acute, subacute, or chronic unilateral myositis or dacryoadenitis, but a wide range of orbital content involvement [4, 5] and clinical presentations are possible, including proptosis, diplopia, conjunctival chemosis, visual disability, restriction of extraocular muscle movement, unilateral periorbital pain, and cranial nerve palsies, typically with dramatic response to corticosteroid therapy [6].

Orbital pseudotumor is commonly restricted to the orbit; however, an extension beyond the orbit can occur, usually in the middle cranial fossa and cavernous sinus, in cases of extensive and chronic inflammation [7], through one of the three major posterior orbital openings: the superior orbital fissure (SOF), the optic canal, and the inferior orbital fissure. Extraorbital extension of orbital pseudotumor in the adjacent paranasal sinuses [8–10], and into the infratemporal and pterygopalatine fossae [11] has also been rarely reported.

Magnetic Resonance Imaging (MRI) is the best technique for imaging orbital pseudotumor, although MRI findings may be nonspecific, and for evaluating its extraorbital extension. The pseudotumor infiltrate in the orbit typically demonstrates low signal intensity on T1-weighted images and frequently on T2-weighted images, depending on the degree of fibrosis, with the sclerosing variety being the most T2-hypointense. Marked gadolinium enhancement is usually present [12]. Orbital pseudotumor may thus mimick several disease entities, including infection, lymphoma, sarcoidosis, and other granulomatous diseases [12, 13]; therefore, it is often a diagnosis of exclusion, based on history, clinical course, response to steroid therapy, laboratory tests, or even biopsy in selected cases.

The intracranial extension of orbital pseudotumor, although rare (8.8 % in a Computed Tomography series), has been previously reported [14–26], and usually involves the middle cranial fossa (MCF) and the cavernous sinus (CS) through the SOF, appearing as an enhancing nodular or plaque-like thickening of the dura mater, as summarized in our literature review (Table 1). In particular, the 3 patterns identified by Clifton et al. [17] still represent the paradigm of the intracranial involvement, eliciting variable edema in the brain tissue contiguous to the extra-axial infiltrate. Here we report the first case, to the best of our knowledge, of orbital pseudotumor presenting with brain MRI findings indicative of intra-axial changes.

**Table 1:** Previously reported cases of orbital pseudotumor with intracranial extension

Year	Author	Patient age (years)	Gender	Intracranial location/features
1984	Kaye et al.	71	M	ACF (planum sphenoidale), dural-based
1986	Frohman et al.	48	M	SOF, optic canal, bone erosion
		48	F	MCF/CS, bone erosion
		72	M	SOF, bone erosion
1986	Noble et al.	46	F	ACF, dural thickening
1992	Clifton et al.	49	M	MCF/CS, pattern II
		69	M	SOF, pattern I
		54	F	MCF/CS, pattern II
		36	M	MCF, pattern I
		86	M	MCF/CS, dural thickening, bilateral involvement, pattern III
		71	F	MCF/CS, pattern II
		30	M	MCF/CS, dural thickening, pattern III
		61	M	MCF/CS, pattern II
1993	Bencherif et al.	23	M	CS, SOF, left fronto-temporal dural thickening, sphenoid bone sclerosis
1993	Olmos et al.	64	F	CS/Meckel cave, parasellar plaque, dural surface down to clivus and C2 body
1996	de Jesus et al.	16	F	Optic canal, SOF, MCF; dural thickening of the left hemisphere and tentorium
1998	Soares et al.	-	-	Pituitary fossa/CS, ICA compression
2000	Ayala et al.	83	F	ACF extra-axial mass without bone involvement, (possibly through the anterior etmoid foramen)
2004	Mahr et al.	40	M	MCF, dural thickening over the temporal pole
		41	M	Optic canal, MCF paraclinoid mass
		73	F	Meckel cave/CS
2005	Lee et al.	58	M	MCF/CS, dural thickening; ICA encasement
		63	M	SOF
		55	M	MCF/CS, petrous apex, dural thickening; brain edema; ICA encasement
		32	M	MCF/CS dural thickening; brain edema
		46	M	MCF/CS, petrous apex, Meckel cave, dural thickening; brain edema; ICA encasement
2006	Zborowska et al.	45	F	MCF/CS, Meckel cave, dural thickening over the temporal pole
		32	M	MCF/CS, pituitary fossa, tentorium; bone erosion (sphenoid wing and orbital roof)
		48	F	MCF/CS; parasellar mass, bone erosion, ICA encasement
2011	Saifudheen et al.	50	M	MCF/CS large dural mass (temporal pole); brain edema

*ACF* Anterior cranial fossa, *MCF* Middle cranial fossa, *CS* Cavernous sinus, *SOF* Superior orbital fissure, *ICA* Internal carotid artery, Pattern I, II, III: patterns of intracranial extension of orbital pseudotumor as described by Clifton et al. [17]

## Case presentation

A 57-year-old obese smoker woman presented with a three-month history of debilitating, daily right-sided headache, worsening with eye movement, right palpebral edema and blurred vision. Her medical history included anxiety, depression and hypertension. Laboratory data showed increased C-reactive protein (5,75 mg/L), erythrocyte sedimentation rate (56 mm/h) and hematic fibrinogen (529 mg/dL). Clinical examination and fundoscopy revealed right conjunctival chemosis, temporal muscle edema, palpebral edema and ptosis, and ill-defined optic disk with papilledema. The visual field and neurological examination were normal.

**Fig. 1:**
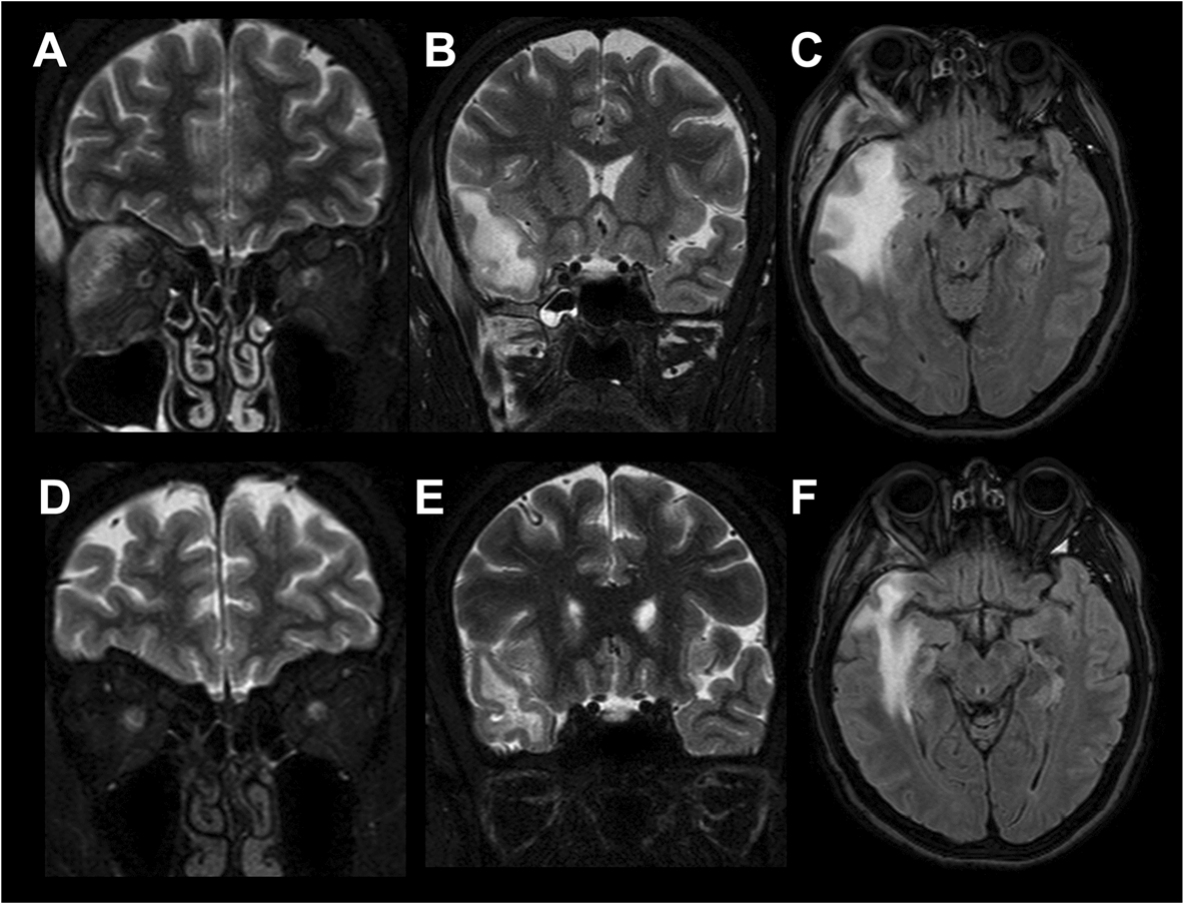
Orbital and extraorbital involvement before and after steroid therapy. Upper row: coronal STIR (**a**, **b**) and axial FLAIR (**c**) MR images before treatment. Lower row: coronal STIR (**d**, **e**) and axial FLAIR (F) MR follow-up images after steroid therapy. Evidence of marked enlargement and edema involving the lateral rectus muscle in the right orbit, with infiltration of the contiguous fat (**a**), and the temporal and pterygoid muscles in the ipsilateral masticator space (**b**), all showing complete regression at follow-up (**d**, **e**). The massive vasogenic edema of the anterior portion of the temporal lobe evident in the initial MR scan (**c**), also shows dramatic reduction at follow-up (**f**)

The patient underwent contrast-enhanced (CE) MRI scan of the brain and orbits, at 1.5 Tesla, with FLAIR (TR: 8005 ms, TE: 100 ms, TI: 2200 ms, matrix: 256x192, slice thickness: 5 mm), STIR (TR: 2650 ms, TE: 90 ms, TI: 180 ms, matrix: 256x204, slice thickness: 3 mm) and Fast Spin-Echo T1- and T2-weighted (TR: 583–4454 ms, TE: 15–100 ms, matrix: 244x194-384x288, slice thickness: 5 mm, respectively) sequences, acquired before and after intravenous administration of Gadobutrol (Bayer HealthCare, 0.1 mmol/Kg). Also, MR angiography of the intracranial arterial and venous systems was performed using 3D and 2D time-of-flight (TOF) sequences, respectively. The MRI scan showed enlargement of the lateral rectus muscle in the right orbit, involving the anterior tendon insertion, infiltration and obliteration of the contiguous fat due to a homogenous T2-hypointense tissue, with marked contrast-enhancement; similar changes were demonstrated in the temporal and the pterygoid muscles within the right masticator space (Fig. 1).

**Fig. 2:**
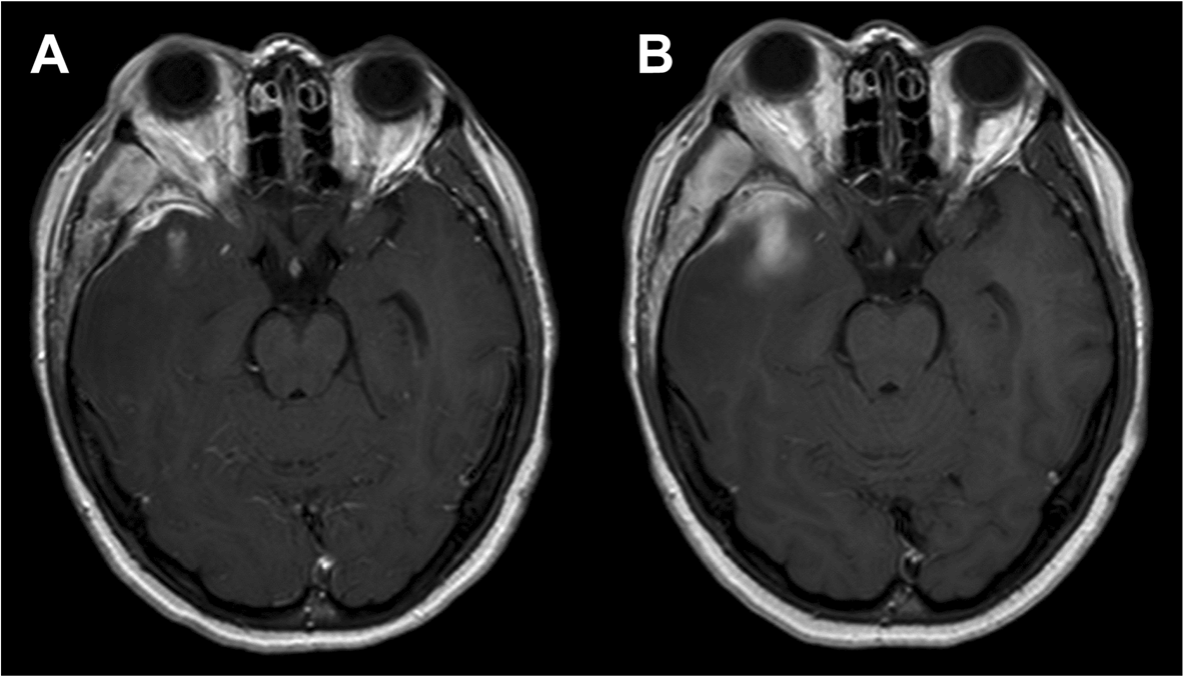
Early and delayed intracranial enhancement in the acute phase. Axial CE T1w MR images before treatment at the level of the middle cranial fossa obtained 5 min (**a**) and 1 h (**b**) after iv administration of Gadolinium. Substantial thickening and intense enhancement of the dura mater adjacent to the right sphenoid wing can be appreciated (**a**), as well as involvement of the sub-cortical white matter of the temporal pole, that increases in the delayed phase (**b**). The temporal muscle also appears markedly enlarged and intensely enhancing compared to the contralateral one

A number of intracranial findings were also detected: massive vasogenic edema of the anterior portion of the right temporal lobe, associated with thickening and enhancement of the contiguous dura (adjacent to the sphenoid wing) and with striking sub-cortical enhancement, that increased at 1 h (Fig. 2). The cavernous sinuses were normal; at MR venography, absence of flow signal in the right spheno-parietal sinus was observed, due to venous compression, with no signs of thrombosis. Also, unenhanced brain Computed Tomography confirmed the intracranial/extracranial edematous changes and disclosed sclerotic reaction of the right sphenoid wing with focal cortical erosion (not shown).

Signs of systemic diseases such as autoimmune disorders were searched using chest x-ray and laboratory tests, including anti-nuclear antibodies, anti-neutrophil cytoplasmic antibodies, C3, C4, and angiotensin-converting enzyme blood levels, all showing no abnormalities. A lumbar puncture was refused by the patient.

Orbital pseudotumor with intracranial involvement was therefore suspected, and the patient was started on steroid therapy (i.v. dexamethasone, 8 mg daily for 4 days, followed by 4 mg for other 4 days, and then tapered with the oral solution).

Biopsy was proposed to the patient as a necessary integration to rule out other possible conditions (e.g. lymphoma) in the acute phase. However, it was firmly refused by the patient.

**Fig. 3:**
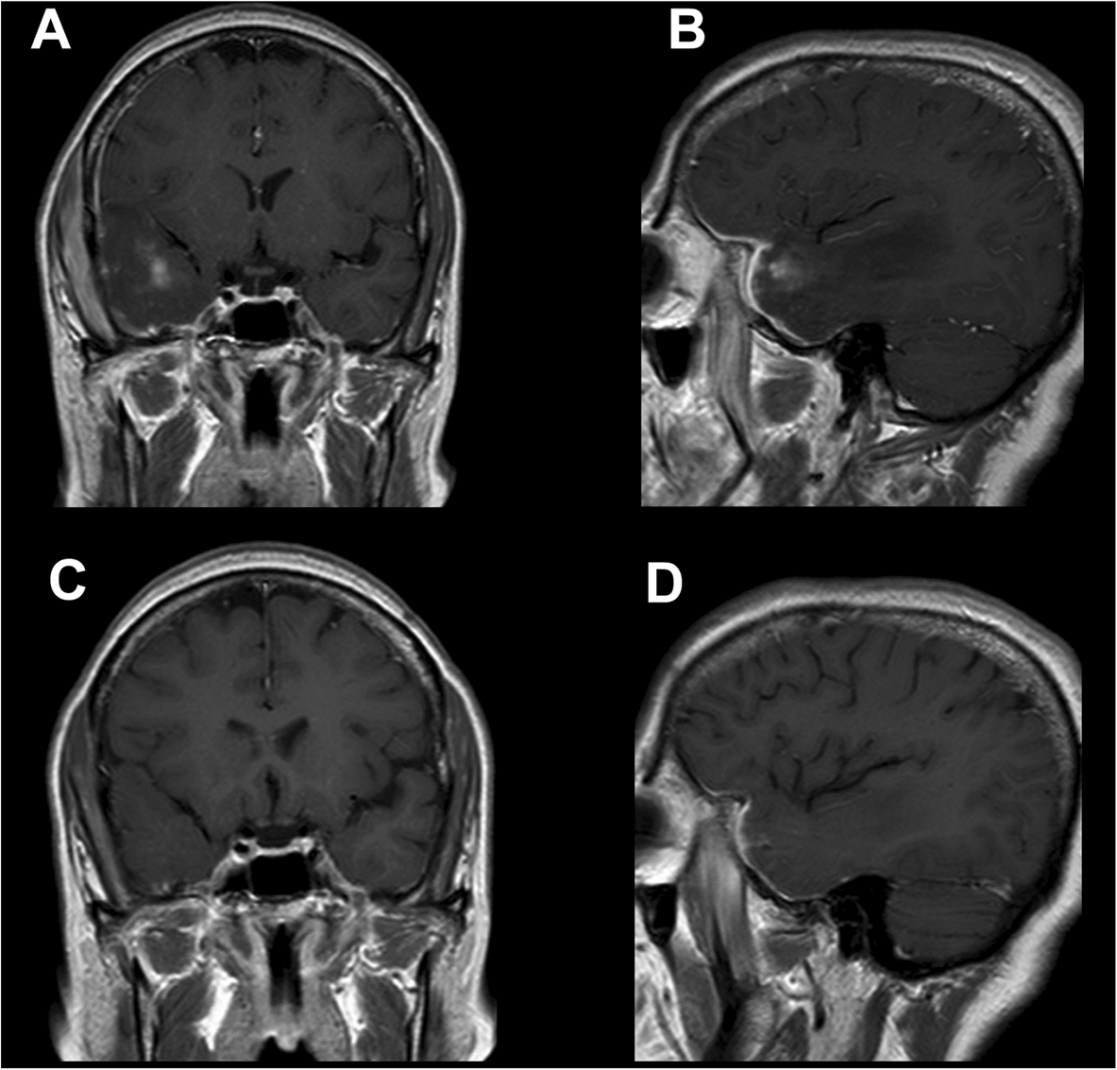
Intracranial enhancement before and after steroid therapy. Coronal (**a**, **c**) and sagittal (**b**, **d**) CE T1w MR images before (upper row) and after (lower row) steroid therapy. In the follow-up phase, CE-MRI (C-D) shows striking improvement of the dural thickening and complete regression of the intra-axial enhancement

Ten days later, follow-up CE-MRI showed dramatic improvement of the intraorbital and extracranial findings, of the dural thickening and of the temporal vasogenic edema, with complete regression of brain enhancement (Figs. 1 and 3), making, at this stage, the biopsy unnecessary. At 6 and 12 month follow-up, the patient continued to do well and her only complaint was mild headache. Further CE-MRI follow-up at 2 years without therapy showed return-to-normal of intraorbital structures, disappearance of intraorbital and intracranial enhancement, and persistence of a small gliotic subcortical scar in the right temporal pole (not shown), reasonably ruling out a diagnosis of lymphoma. The patient is still periodically seen in our Institute (now at 2.5 years), and reports no further episodes of orbital swelling nor headache.

In our patient, the subcortical temporal pole enhancement, which increased in the delayed phase (Fig. 2), represented the intra-axial consequences of the intracranial orbital pseudotumor extension, which, to our knowledge, have never been previously described.

In our opinion, a possible pathophysiological explanation of the observed findings may take origin from the compression of the loco-regional venous system, due to the meningeal extension of the pseudotumor, even if small, as demonstrated by the impaired visualization of the right spheno-parietal sinus at MR venography. It is known that cerebral venous thrombosis could lead to both vasogenic edema and brain parenchymal enhancement [27], which can be either reversible or irreversible [28], allowing us to hypothesize the presence of sinus venous compression in the present case, with pathophysiological consequences similar to vein thrombosis. A state of venous hypertension of the cortical veins of the temporal pole, without actual thrombosis (that was not present in any of the CE-MRI scans), may thus have led to hampered venous drainage, with damage of the blood–brain barrier with increased permeability and CE in the temporal lobe ensuing as a functional consequence, which resolved after successful therapy (Fig. 3). The associated vasogenic edema was therefore due to the venous hypertension and not to the orbital pseudotumor mass itself, as also supported by the discrepancy between the small actual bulk of the intracranial pseudotumoral tissue and the large extent of intra-axial vasogenic edema (Figs. 1, 2 and 3).

We have reviewed all orbital pseudotumor cases with intracranial extension that we could retrieve in the literature (Table 1). Compared to our patient, most cases exhibited somewhat similar features, such as the extra-axial involvement of MCF – CS, and mild vasogenic edema as the only intra-axial finding. However, none of them showed intra-axial enhancement and large subcortical edema, thus mimicking intra-axial pathology, which, instead, were demonstrated in our case using delayed CE-MRI acquisitions. We could also document a complete regression of the intra/extra-cranial findings, due to serial MRI scans over a 2-year follow-up, not available in most other reported cases.

## Conclusions

In conclusion, this case serves as a reminder that chronic inflammatory orbital pseudotumor can extend intracranially, possibly inducing serious neurological symptoms, and even showing MRI changes mimicking intra-axial pathology. Although invasive orbital pseudotumors are uncommon, they should be considered in the differential diagnosis of orbital masses extending beyond the confines of the bony orbit. CE-MRI is the method of choice to evaluate both extracranial and intracranial pathology, possibly with delayed acquisitions, and may help, along with biopsy, in recognizing these rare findings and in monitoring the appropriate therapy.

## Consent

Written informed consent was obtained from the patient for publication of this Case report and any accompanying images. A copy of the written consent is available for review by the Editor of this journal.

## Abbreviations

ACF: Anterior cranial fossa
CE-MRI: Contrast-enhanced MRI
CS: Cavernous sinus
CT: Computed tomography
FLAIR: Fluid attenuated inversion recovery
ICA: Internal carotid artery
MCF: Middle cranial fossa
MRI: Magnetic resonance imaging
SOF: Superior orbital fissure
STIR: Short tau inversion recovery
TE: Echo time
TI: Inversion time
TR: Repetition time
